# Supplementary material for: Serum presepsin level reflects macrophage activation and hemophagocytosis in bone marrow
Source: PLoS One. 2026 Apr 1;21(4):e0344867. doi: 10.1371/journal.pone.0344867 (PMC13042860; doi:10.1371/journal.pone.0344867)
Supplement: S1 Fig — IHC was performed using CD68 (PG-M1) and CD14 (D7A2T) antibodies. Panels (a–c) show bone marrow findings before HLH onset: (a) hematoxylin and eosin (H&E) staining, (b) CD68, and (c) CD14. Panels (d–f) show bone marrow findings after HLH onset: (d) H&E, (e) CD68, and (f) CD14. Arrowheads indicate phagocytic cells. Scale bar = 50 μm. (PDF) [file pone.0344867.s001.pdf]

Supplemental Figure1

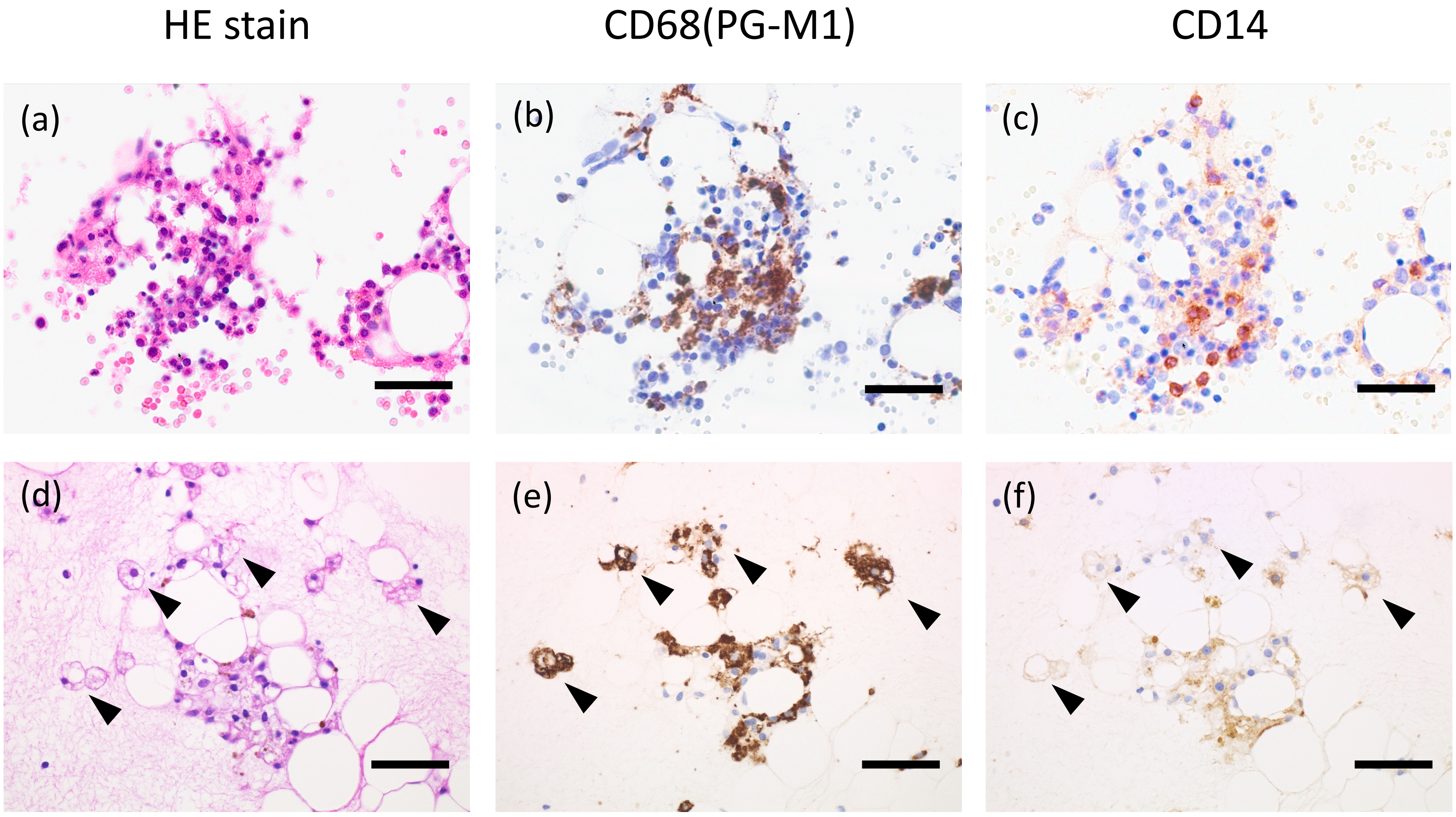

Comparison of macrophage marker and presepsin precursor protein expression in the bone marrow before and after the onset of HLH.

IHC was performed using CD68 (PG-M1) and CD14 (D7A2T) antibodies. Panels (a–c) show bone marrow findings before HLH onset: (a) hematoxylin and eosin (H&E) staining, (b) CD68, and (c) CD14.Panels (d–f) show bone marrow findings after HLH onset: (d) H&E, (e) CD68, and (f) CD14.

Arrowheads indicate phagocytic cells. Scale bar = 50 µm.
