# Supplementary material for: Serum presepsin level reflects macrophage activation and hemophagocytosis in bone marrow
Source: PLoS One. 2026 Apr 1;21(4):e0344867. doi: 10.1371/journal.pone.0344867 (PMC13042860; doi:10.1371/journal.pone.0344867)
Supplement: S1 Table — (DOCX) [file pone.0344867.s002.docx]

**S1 Table. Frequencies of findings based on the diagnostic criteria for hemophagocytic lymphohistiocytosis.**

|  | Number of patients (%) |
| --- | --- |
| Persistent fever  (lasting 7 days or more, with a peak of 38.5 °C or higher) | 7 / 61 (11.5 %) |
| Cytopenia (Two or more lineages in peripheral blood) | 9 / 61 (14.8 %) |
| Anemia (≤ 9 g/dL) | 10 / 61 (16.4 %) |
| Thrombocytopenia (≤ 100×10^9^ /L) | 15 / 61 (24.6 %) |
| Neutropenia (≤ 1×10^9^ /L) | 7 / 61 (11.5 %) |
| Hyperferritinemia  (> 1000 ng/mL or at least the mean plus 3 standard deviations of the age-appropriate normal range) | 11 / 61 (18.0 %) |
| Elevated lactate dehydrogenase  (> 1000 U/L or at least the mean plus 3 standard deviations of the age-appropriate normal range) | 2 / 61 (3.3 %) |
| Pathological hemophagocytosis in bone marrow, spleen, or lymph nodes | 46 / 61 (75.4 %) |
| Number of patients meeting all HLH diagnostic criteria | 1 / 61 (1.6 %) |

HLH, hemophagocytic lymphohistiocytosis.
