# Supplementary material for: Serum presepsin level reflects macrophage activation and hemophagocytosis in bone marrow
Source: PLoS One. 2026 Apr 1;21(4):e0344867. doi: 10.1371/journal.pone.0344867 (PMC13042860; doi:10.1371/journal.pone.0344867)
Supplement: Attachment — Submitted filename: PONE-D-25-46766_reviewer comments.pdf [file pone.0344867.s003.pdf]

Reviewer comments to the author **Date: 18 September, 2025**

PONE-D-25-46766

Title: Serum presepsin levels reflect macrophage activation and hemophagocytosis in bone marrow

The manuscript submitted by Katsumasa Yamanaka et al. provides an important contribution to the scientific community and other concerned bodies regarding the correlation between Serum presepsin levels and hemophagocytosis in bone marrow. However, certain revisions are required to the submitted manuscript.

Major Comments

Abstract

Page 5, line 2: background is more preferable than objective, but include your objective under the heading of background.

Introduction:

1. Page 7, Line 16: space between the word "blood" and the reference.

Methods and materials

1. *Participants is more formal than patients if possible.*

2. *How many of your study participants are with HLH*

3. Page 9, line 4-5: Patients with an estimated glomerular filtration rate (eGFR) <30 (mL/min/1.73 m^2) were excluded……. *Needs citation (reference).*

4. Page 9, line 14: Serum samples were collected from residual patient samples. *What was the tube used to collect the blood, at what centrifugal force was the blood centrifuged to separate the serum?* ***Please state this section in detail.***

5. Bone marrow samples were obtained *from patients with B-cell lymphoma without bone marrow infiltration and from patients with HLH after bone marrow transplantation*. Lymph node samples were collected from *patients with lung cancer or B cell lymphoma*. *Why you have collected bone marrow and lymph node samples only from those study participants since there are other hematological and autoimmune diseases included in your result section (table 1)?* ***State clearly please?***

6. You know that in histopathology lab, there are certain processes that the tissue section should undergo before mounting including tissue processing and staining. *Thus, more detailed information is needed.*

7. Page 11, Line 10: Data are expressed as mean ± standard deviation (SD). *Are all your data continuous and normally distributed? Additionally, were is more preferable than are*

Result

1. Page 1, Line 10-12: LD, Ferritin and presepsin are singular. Thus, say *level,* not *levels* and was, not *were or* you can state as *"The LD, Ferritin and presepsin levels were increased with their respective concentration of …….*

2. Page 12, Line 14: D-dimer levels were elevated at 6.0 ± 9.0 µg/mL, as well as soluble fibrin (15.5 15 ± 23.6 µg/mL). *Rewrite it* like *D-dimer as well as soluble fibrin levels were elevated (6.0 ± 9.0 µg/mL and 15.5 15 ± 23.6 µg/mL respectively).*

3. Page 12, Line 15-16: Bone marrow findings showed an *increased nucleated cell count* (77.8 ± 16 182.3×10⁹/L) and an *elevated percentage of hemophagocytes* (7.5 ± 12.7%). *Define elevated nucleated cell and pathological percentage of hemophagocytes in bone marrow (i.e. need operational definitions).*

4. Page 13, Line 3: In 61 patients, the *median serum presepsin* level was 311.2 pg/mL (range 91-1895 pg/mL). It is preferred to say "*in this study, the median serum presepsin level was 311.2 pg/mL (range 91-1895 4 pg/mL). Additionally, in your method and material section under" Statistical analysis*" you have stated that "Data *are expressed as mean ± standard deviation (SD)*". *Here you stated the serum presepsin level in median (range), but in mean± SD (303.8 ± 288.8) as shown in table 2 and page 12, line 12* (*check your analysis again please*).

5. Page 13, Line 10: Additionally, presepsin *level was* significantly correlated with bone marrow findings, including……, Not Additionally, presepsin *levels* significantly correlated with bone marrow findings, including……

6. Page13, Line 15-16: Presepsin precursor CD14 protein expression is attenuated in hemophagocytes in lymph nodes and bone marrow. No need of full stop (.) at the end since it is a sub-title.

7. Page 14, Line 4-6: In the normal bone marrow……………………..within the normal ranges. *Support it with figure cross reference (fig…..)*

8. Page14, Line 6-7: However, in bone marrow with hemophagocytosis, *blood cells numbers were reduced, while macrophage numbers were increased. Please determine what proportion of your study participants have reduced RBC, WBC (leukopenia) and platelet count (thrombocytopenia) since HLH is characterized by cytopenia….. (Introduction… line 6)*

Discussion

1. Page 15, Line 2: "In this study, we investigated serum and bone marrow smear specimens from 61 patients and found a correlation between serum presepsin levels and macrophage activation in the bone marrow, particularly during hemophagocytosis". ***Paraphrase it again like*** *" In this study, we have investigated the correlation between serum presepsin level and macrophage activation in the bone marrow, particularly during hemophagocytosis"*.

2. Page 15, Line 17: Previous studies……. Implies more than one study, but you have cited only one study (18).

3. Page 16, line 15-17: "Despite our small sample size, we found a strong correlation between serum markers of 16 hemophagocytosis, β2-microglobulin (n=21, r=0.782, p<0.001) and sIL-2R (n=15, 17 r=0.689, p=0.006), and the ratio of phagocytes in the bone marrow of patients with HLH". *Please cite it if you have published it or state as "**result not indicated**".*

4 .page 16, 5-8: *"*In addition, since the expression of CD68 on macrophages does not change during both steady state and hemophagocytosis, a decrease in the ratio of CD14 positive cells/CD68 positive cells in macrophages may be used as an indicator of hemophagocytes". Therefore, *if possible, include the ratio of CD14+/CD68+ cells before and after hemophagocytosis in your result section.*

Finally, conclude your study standing from your finding and forward your recommendations to the concerned body especially for those diagnosing HLH solely on hemophagocytes

Minor comments

1. Check your grammar.

Good luck!
