# Supplementary material for: Serum presepsin level reflects macrophage activation and hemophagocytosis in bone marrow
Source: PLoS One. 2026 Apr 1;21(4):e0344867. doi: 10.1371/journal.pone.0344867 (PMC13042860; doi:10.1371/journal.pone.0344867)
Supplement: Attachment — Submitted filename: PONE-D-25-46766_R2 comments.pdf [file pone.0344867.s007.pdf]

PONE-D-25-46766R2

Title: Serum presepsin levels reflect macrophage activation and hemophagocytosis in bone marrow

Comments to the Author

No comments.
