# Supplementary material for: Serum presepsin level reflects macrophage activation and hemophagocytosis in bone marrow
Source: PLoS One. 2026 Apr 1;21(4):e0344867. doi: 10.1371/journal.pone.0344867 (PMC13042860; doi:10.1371/journal.pone.0344867)
Supplement: Attachment — Submitted filename: renamed_6ba7e.docx [file pone.0344867.s008.docx]

**RESPONSE TO REVIEWER #2**

**1. Page 14 line 8: Re-write it. “In this study, the serum presepsin level was mean 303.8 ± 288.8 pg/mL, and median 222 pg/mL (IQR 154-313 pg/mL).” Like as “In this study, the mean serum presepsin level was 303.8 ± 288.8 pg/mL, and that of its median was 222 pg/mL (IQR 154-313 pg/mL)”.**

We have addressed the point the reviewer mentioned.

**2. Please ensure proper use of subject-verb agreement throughout your document. For instance, page 17 line 1-4 “Presepsin level in sepsis patients, which are also commonly used in clinical practice, have been reported as 817.9 ± 572.7 pg/mL in sepsis patients and 1,992.9 ± 1,509.2 pg/mL in severe sepsis patients, compared to 294.2 ± 121.4 pg/mL in healthy individuals”. This statement refers to Presepsin level, not septic patients. Thus, are should be replaced by is and have been by has been, similarly line 10… Elevated presepin level in sepsis are primarily attributed….**

We apologize for the incorrect description. We have addressed the point the reviewer mentioned.
