# Supplementary material for: Serum presepsin level reflects macrophage activation and hemophagocytosis in bone marrow
Source: PLoS One. 2026 Apr 1;21(4):e0344867. doi: 10.1371/journal.pone.0344867 (PMC13042860; doi:10.1371/journal.pone.0344867)
Supplement: Attachment — Submitted filename: PONE-D-25-46766_R1 Reviewer comments.pdf [file pone.0344867.s005.pdf]

Date: 18 December, 2025

PONE-D-25-46766R1

Title: Serum presepsin levels reflect macrophage activation and hemophagocytosis in bone marrow

Comments to the Author

Dear Authors,

Thank you for your revisions. The manuscript has been significantly improved as a result of your efforts. That said, I still have a few remaining minor comments that require your attention.

1. Page 14 line 8: Re-write it. “In this study, the serum presepsin level was mean 303.8 ± 288.8 pg/mL, and median 222 pg/mL (IQR 154-313 pg/mL).” Like as “In this study, the mean serum presepsin level was 303.8 ± 288.8 pg/mL, and that of its median was 222 pg/mL (IQR 154-313 pg/mL)”.

2. Please ensure proper use of subject-verb agreement throughout your document. For instance, page 17 line 1-4 “Presepsin level in sepsis patients, which are also commonly used in clinical practice, have been reported as 817.9 ± 572.7 pg/mL in sepsis patients and 1,992.9 ± 1,509.2 pg/mL in severe sepsis patients, compared to 294.2 ± 121.4 pg/mL in healthy individuals”. This statement refers to Presepsin level, not septic patients. Thus, are should be replaced by is and have been by has been, similarly line 10… Elevated presepin level in sepsis are primarily attributed….

Best regards,
